# Staphylococcal Bicomponent Pore-Forming Toxins: Targets for Prophylaxis and Immunotherapy

**DOI:** 10.3390/toxins6030950

**Published:** 2014-03-04

**Authors:** M. Javad Aman, Rajan P. Adhikari

**Affiliations:** Integrated BioTherapeutics Inc., 21 Firstfield Rd., Gaithersburg, MD 20878, USA; E-Mail: rajan@integratedbiotherapeutics.com

**Keywords:** *Staphylococcus aureus*, pore-forming toxin, leukotoxins, leukocidins, hemolysin, vaccine, immunotherapy, PVL, LukAB, LukED, HlgAB, HlgCB

## Abstract

*Staphylococccus aureus* represents one of the most challenging human pathogens as well as a common colonizer of human skin and mucosal surfaces. *S. aureus* causes a wide range of diseases from skin and soft tissue infection (SSTI) to debilitating and life-threatening conditions such as osteomyelitis, endocarditis, and necrotizing pneumonia. The range of diseases reflects the remarkable diversity of the virulence factors produced by this pathogen, including surface antigens involved in the establishment of infection and a large number of toxins that mediate a vast array of cellular responses. The staphylococcal toxins are generally believed to have evolved to disarm the innate immune system, the first line of defense against this pathogen. This review focuses on recent advances on elucidating the biological functions of *S. aureus* bicomponent pore-forming toxins (BCPFTs) and their utility as targets for preventive and therapeutic intervention. These toxins are cytolytic to a variety of immune cells, primarily neutrophils, as well as cells with a critical barrier function. The lytic activity of BCPFTs towards immune cells implies a critical role in immune evasion, and a number of epidemiological studies and animal experiments relate these toxins to clinical disease, particularly SSTI and necrotizing pneumonia. Antibody-mediated neutralization of this lytic activity may provide a strategy for development of toxoid-based vaccines or immunotherapeutics for prevention or mitigation of clinical diseases. However, certain BCPFTs have been proposed to act as danger signals that may alert the immune system through an inflammatory response. The utility of a neutralizing vaccination strategy must be weighed against such immune-activating potential.

## 1. Introduction

*Staphylococcus aureus* (*S. aureus*) is a ubiquitous, formidable Gram-positive microorganism that acts both as a human commensal, as well as a pathogen. *S. aureus* is associated with a wide range of diseases from skin and soft tissue infections to life-threatening systemic disease and is a leading cause of hospital-associated (HA) and community-associated (CA) infections worldwide [1,2,3,4]. The range of diseases reflects the diverse abilities of this microbe to escape the innate and adaptive immune response using multiple virulence factors, including coagulase; capsular polysaccharides; adhesins; proteases; exoproteins that inactivate the complement system; pore-forming toxins; superantigens; and other innate response mediators [1,5]. The problem is exacerbated by increasing prevalence of methicillin-resistant *S. aureus* (MRSA), making the development of vaccines and immunotherapeutics for this pathogen a pressing public health need.

Initially MRSA strains were mainly limited to healthcare settings; however, over the past two decades several epidemics of community-associated MRSA (CA-MRSA) have been reported that cause severe disease in an otherwise healthy population. To date, five CA-MRSA clones are associated with these outbreaks: the Midwest clone (MW2 USA400), the European clone, the Southwest–Pacific Oceania clone, the Pacific clone, and the Pandemic clone (USA300), belonging to the clonal complexes 1, 80, 30, 59, and 8, respectively [6,7,8]. In addition to SCC*mec* IV, a prominent characteristic of these major CA-MRSA clones is that they all have the *luk*PV operon encoding the Panton Valentine Leukocidin (PVL) [9], carried by the lysogenic phages ϕSLT, ϕPVL, ϕSA2MW and ϕSA2usa [10,11,12]. This observation renewed interest in this pore-forming bicomponent toxin which was first described by Panton and Valentine in 1932 [13]. As a phage-encoded toxin, PVL is expressed only in 2%–4% of *S. aureus* clinical isolates [14,15,16]. Since the association of PVL with severe necrotizing pneumonia and skin infections was postulated [15,17,18,19,20] the role of this toxin as a key virulence factor of *S. aureus* in the pathogenesis of CA-MRSA has been a hot topic of debate. Conflicting results have been reported that show a role for PVL in pathogenesis [15,17,18,21,22,23], no role [24,25], or even reduced virulence [26,27] depending on the experimental setting or animal models used. Recent discovery of a PVL receptor and reports on its tropism and species specificity have shed new light on some of the conflicting observations. Nevertheless, the focus on PVL has largely distracted the scientific community from other bicomponent pore-forming toxins (BCPFTs) with high sequence identity to PVL that are, in contrast to PVL, chromosomally encoded and expressed in a wide range of clinical isolates [16]. A number of excellent reviews have been published on the genetics and structure of bicomponent pore-forming toxins [28,29,30]. In this review, we focus on recent advances on understanding of the cellular receptors, tropism, and biological activities of these toxins as they relate to the potential utility of this family of toxins as therapeutic and prophylactic targets.

## 2. Staphylococcal Bicomponent Pore-Forming Toxins (BCPFTs)

*S. aureus* produces three classes of cytolytic toxins: (i) the short amphiphilic peptides including delta toxin [31], and phenol soluble modulins (PSM) [32]; (ii) single component alpha hemolysin (Hla; α-toxin) [33], and bicomponent leukotoxins including leukocidins and gamma hemolysin (Hlg) [28]. While PSM and delta toxin are inserted into membranes by the virtue of their amphiphilic nature, the pore-forming Hla, leukocidins, and Hlg require oligomerization to form functional pores, a process that requires binding to specific cell surface receptors. Hla monomers assemble into a heptameric pre-pore structure at the plasma membrane followed by formation of the pore. The bicomponent pore-forming toxins (BCPFTs), consist of two subunits with a beta barrel structure that acquire pore-forming conformation upon binding to specific cellular receptors followed by hetero-oligomerization at the plasma membrane of the target cells. The oligomeric toxins then insert a pore into the plasma membrane leading to ion influx and efflux, initiation of a variety of apoptotic and necrotic processes, and ultimately cell death.

The BCPFTs consist of two classes of proteins denoted as S and F subunits [28]. Each subunit is produced and secreted separately and the association does not occur until the binding to the cell surface receptor is initiated. Current data indicate that the S subunit is the primary receptor binding subunit [34,35,36,37,38]. Upon binding of the S subunit to its cellular receptor it forms a heterodimer with the F component followed by multimerization leading to a ring-like structure of ~200 KDa with an external diameter of 7–9 nm and internal diameter of 2–3 nm on red blood cells and polymorphonuclear cells (PMN) [39,40]. While the pore-forming conformation of Hla consists of homo-heptamers [41], crystal structure and modeling studies have proposed heptameric [42], hexameric [43] and octameric [44,45,46] structures for the functional BCPFT complexes. For bicomponent toxins, a heptameric structure is very unlikely given the lack of symmetry and the fact that, in such an arrangement at least one interaction per oligomer must occur between identical subunits. The currently accepted model consists of four S and four F subunits assembled into an octamer with an alternating arrangement [46]. Insertion of pores into the plasma membrane results in osmotic lysis of target cells [28]. While this direct cell lysis has been considered the primary pathophysiological function of these toxins, recent studies indicate that BCPFTs are able to trigger a complex array of cellular processes that can significantly impact the innate immune response to *S. aureus* [26,29,30,47].

The leukocidin family of BCPFTs includes the chromosomally encoded LukED, LukAB, as well as the phage-encoded Pantone–Valentine leukocidin (PVL). PVL subunits LukS-PV and LukF-PV are encoded by the *luk*PV operon [9], carried by the lysogenic phages ϕSLT, ϕPVL, ϕSA2MW and ϕSA2usa [10,11,12]. A PVL-like operon has been also reported to be formed by LukM/LukF-PV(P83) in the genome of the prophage *ϕP83-pro* [48]. LukM/LukF-PV(P83) has been proposed as a virulence factor in mastitis due to sensitivity of ruminants’ leukocytes to this toxin [49]; however, a recent report shows the inability of this toxin to induce inflammation in the udder [50]. The association of PVL with the five major pandemic clones of CA-MRSA led to the hypothesis that PVL is the key virulence factor for CA-MRSA necrotizing pneumonia and SSTI [51,52,53]. Epidemiological association between LukED and human disease has been evaluated in a few recent reports. Among other virulence factors, significant association has been reported between LukED expression and *S. aureus* invasive disease [54,55]. Such association also has been shown for skin dermatitis and furuncles from HIV+ patients [56], impetigo [57], as well as *S. aureus*-associated diarrhea [58]. Epidemiological studies evaluating association of LukAB with human disease have not been reported. However, a recent report indicates strong neutralizing antibody response to LukAB in pediatric patients with *S. aureus* invasive disease [59]. A role for LukED [60] and LukAB [61] in pathogenesis also has been shown in mouse models of *S. aureus* infection.

Gamma hemolysins include alternative combinations of two S subunits HlgA or HlgC with the F subunit HlgB. HlgAB and HlgCB are reported to lyse human and other mammalian erythrocytes [28], PMNs [62,63], and to enhance the survival of *S. aureus* in human blood [64].

While the role of PVL as CA-MRSA virulence factor remains controversial, an often ignored fact is the extensive sequence identity within the leukotoxin family all of which, except for PVL, are chromosomally encoded and expressed in most strains. It is therefore critically important to extend the epidemiological and biological studies to the entire family of leukocidins. This is now substantially facilitated by recent discovery of several receptors for these toxins.

## 3. Tropism and Species Specificity

A summary of the reported cell type specific activities and species specificities of BCPFTs is shown in Table 1. While the tropism of BCPFTs is largely restricted to hematopoietic cells, individual toxins show distinct and overlapping cell tropism, as well as species specificity. Furthermore, it is important to note that the observed tropism may be dependent on the functional read-out studied. Polymorphoneuclear phagocytes (PMN) are the most prominent target of BCPFTs as these cells are lysed by PVL, LukED, LukAB, and Hlg. Lysis of red blood cells has been traditionally examined in the studies related to hemolysins. However, as noted in this review several leukocidins are hemolytic and hemolysins can kill neutrophils. Therefore, the species specificity and tropism can be different based on whether toxicity towards leukocytes or erythrocytes is considered.

**Table 1:** Cellular tropism and species specificity of BCPFTs.

Cells and Species	Toxin	Effect	References
Human PMN	PVL	Receptor binding	[36,38]
		Lysis	[36,38,65,66]
		Ca++ influx	[36,67]
		Proinflammatory response	[38,66]
	LukED	Lysis	[63,68,69] and Figure 1
		Receptor binding	[68]
		Ca++ influx	[69,70]
		Proinflammatory response	[69]
	LukAB (LukGH)	Receptor binding	[71]
		Lysis	[61,66,72,73,74]
	HlgAB/CB	Lysis	[63] and Figure 1
Murine PMN	PVL	No effect	[65,66]
		Proinflammatory response	[26,75]
	LukED	Lysis	[34,60]
	LukAB	No effect	[71]
		Lysis	[72]
Rabbit PMN	PVL	Lysis	[19,66]
		Proinflammatory response	[66]
	LukAB	Lysis	[72]
Human Monocytes and Macrophages	PVL	Receptor binding	[36,38]
		Proinflammatory response	[76]
	LukAB LukED	Lysis	[61]
		Lysis	[34,68]
Human T cells	LukED	Lysis	[34]
Human Natural Killer cells	LukED	Lysis	[68]
Murine Monocytes and Macrophages	PVL	Proinflammatory response	[75]
	LukED	Lysis	[60]
Human DC	LukAB	Lysis	[61]
	LukED	Lysis	[34]
Human RBC	Hlg	Lysis	[28]
Rabbit RBC	Hlg	Lysis	[11,39,77,78]
	LukED	No effect	[79]
		Lysis	[80] and Figure 1

The cytolytic activity of PVL appears to have a very strict species specificity only affecting human and rabbit cells with no lytic activity towards mouse and even nonhuman primates (NHPs) [66]. While robust lysis of monocytes and macrophages by PVL has not been reported, two reports show binding of LukS-PV to human monocytes and macrophages [36,38]. In addition, induction of proinflammatory cytokines in human [76] and murine macrophages [75] by PVL has been reported. In contrast, LukED appears to have a broader range of cytolytic activities towards both myeloid and T cell lineages as it kills human PMN, monocytes-derived macrophages, dendritic cells, as well as effector memory T lymphocytes [34]. LukED is also cytolytic towards murine PMN [34,60]. The original report of cloning LukED describes this toxin as a dermonecrotic toxin in rabbits with no hemolytic activity [79]. However, Morinaga *et al*. showed that LukED is cytotoxic to both rabbit leukocytes as well as erythrocytes [80]. To avoid confusion, it must be noted that the LukE and LukD sequences reported by Morinaga *et al*. that were perceived to be a variant (LukEDv) indeed represent the conserved sequences of LukE and LukD toxins.

LukAB (also called LukGH [73]) is the most divergent BCPFT based on sequence homology with other leukotoxins. Similar to LukED and PVL, LukAB is lytic towards human PMN [61,66,71,72,73], as well as NHP and rabbit PMN [72]. LukAB also kills human macrophages and dendritic cells [61]. Dumont *et al*. reported lack of binding of LukAB to the murine counterpart of its receptor [71]. Another report indicates that LukAB is cytolytic towards murine PMN; however, this activity was ten and thousand-fold lower than rabbit and human PMNs, respectively [72]. Activity of LukAB towards murine cells is consistent with another report by Dumont *et al*. showing a role for LukAB in a murine renal abscess model of *S. aureus* infection [61].

Gamma hemolysins (HlgAB and HlgCB) have been described based on their hemolytic activity [81]. This bicomponent toxin forms cation-sensitive pores [82] of 2.1–2.4 nm in the plasma membrane [83] and causes osmotic lysis in human erythrocytes [77,78]. HlgAB has been shown to lyse human and rabbit erythrocytes as well as human PMN [62]. Malachowa *et al*. reported that HlgA, B, and C were upregulated when USA300 was grown in human blood [64], and this correlated with increased bacterial survival in the blood. An isogenic *hlgABC*-deletion strain (LAC*ΔhlgABC*) showed a significantly reduced capacity to lyse human neutrophils and to induce lethality in a mouse bacteremia model [63]. Similar to PVL, HlgAB has been shown to be dermonecrotic in rabbits [84]. Hlg components A, B, and C are shown to form mixed oligomeric pore-forming structures leading to a larger than expected number of active toxins by cross-combining various S and F components [82].

## 4. Cellular Receptors

Recently, cellular receptors for PVL, LukAB, and LukED have been identified demonstrating that these toxins exploit key surface proteins important for a variety of immune cell functions. Spaan *et al.* tested the ability of PVL subunits to inhibit binding of monoclonal antibodies to 56 different cell surface receptors and determined that LukS-PV interfered with the binding of three antibodies to human complement C5a receptor (C5aR) [38]. In contrast, LukF-PV did not inhibit the binding of the antibodies consistent with previous findings that the S subunit is the primary cell binding component [38]. Further binding assays showed that LukS-PV directly binds to C5aR as well as the closely related receptor C5L2 with low nanomolar EC_50_ values. Both of these receptors are expressed on neutrophils and monocytes but not on lymphocytes [38,85,86], consistent with the described tropism of PVL [36]. HEK 293 cells transfected with murine or macaque C5aR did not bind to LukS-PV, while transfection with human or rabbit counterparts mediated LukS-PV binding [38], providing a clear explanation for the reported species specificity of PVL [65,66]. PVL-induced lysis was inhibited by competition with purified C5a in neutrophils or silencing of C5aR in THP-1 macrophage cells, demonstrating that this interaction is required for the functional pore formation [38]. Furthermore, priming of neutrophils by sublytic concentrations of PVL was mediated by C5aR [38].

Complement C5a activation of C5aR is an important step in sensing bacterial infection by phagocytes. Interestingly, binding of LukS-PV to C5aR inhibited the activation of neutrophils by C5a, suggesting that LukS-PV alone may mediate immune evasion via this route. However, since LukS-PV gene expression is always accompanied by LukF-PV gene expression, it is not clear if this inhibitory activity in the absence of cytolysin formation plays a meaningful role in *S. aureus* pathogenesis.

Alonzo *et al*. recently reported the involvement of yet another G protein-coupled receptor in mediating the activity of staphylococcal leukocidins. The HIV co-receptor CCR5 was shown to mediate binding and cytolysin formation by LukED [34]. In contrast to CCR5-deficient Jurkat cells, CCR5-expressing HUT-R5 T cells were killed by LukED, and this cytotoxic activity was reduced after treatment of the cells with CCR5 antagonist Maraviroc. LukED killed human macrophages and monocyte-derived dendritic cells, and this killing was also inhibited by Maraviroc [34]. Ectopic expression of CCR5 also rendered CCR5 negative cells sensitive to LukED (but not to LukAB or PVL). Direct binding of CCR5 was shown to be mediated by LukE [34], consistent with the notion that the S subunits are primary cell binding components of BCPFTs [28]. Lymphocytes and macrophages from CCR5^−/−^ mice were resistant to LukED cytotoxicity [34]. On the other hand, LukED can also kill monocytes and PMN in a CCR5-independent manner, thereby suggesting that other receptors may be also involved [60,79,80]. Indeed, Reyes-Robles *et al.* more recently reported that the chemokine receptors CXCR1 and CXCR2 serve as LukE receptors on neutrophils, monocytes, and natural killer (NK) cells [68].

Targeting of T lymphocytes through CCR5, a unique property of LukED, appears to have important functional consequences for the adaptive immune response. Treatment of human peripheral blood mononuclear cells with LukED resulted in depletion of memory T lymphocytes [34] suggesting a role for LukED in evasion of *S. aureus* from long-lasting adaptive immune response. Interestingly, LukED treatment reduced the frequency of IL-17 producing cells, an arm of cell-mediated immunity recently reported by several groups to be important for protection against *S. aureus* [87,88,89,90].

These data indicate that LukED has suppressive effects on both innate and adaptive arms of the immune system. However, LukED appears to be a much less potent toxin than PVL, as PVL toxicity is evident at low and sub-nanomolar concentrations [38], while detectable LukED toxicity requires concentrations above 30–100 nM [34]. On the other hand, CCR5^−/−^ mice challenged with a LukED expressing *S. aureus* strain showed significantly higher survival compared to wt counterparts [34], suggesting that relevant concentrations of LukED can be achieved *in vivo* during infection.

CD11b, the alpha subunit of αM/β2 (CD11b/CD18) integrin receptor has been identified as cellular receptor for LukAB on human PMNs [71]. LukAB binds with a dissociation constant (*K*d) of about 38 nM to human CD11b, whereas binding to the murine counterpart was very poor with *K*d above 10 mM [71]. This species specificity appears to relate to binding of LukAB to the I domain of CD11b which is divergent between human and mouse proteins. Down-regulation of CD11b on HL-60-derived PMNs reduced sensitivity to LukAB, but not to PVL, and ectopic expression of CD11b rendered HEK 293 cells sensitive to LukAB [71].

The initial report by Dumont *et al.* [71] does not specify which component of LukAB is responsible for binding to CD11b as all binding experiments and affinity measurements were performed with the full bicomponent toxin. This was clarified by a more recent publication by this group showing that LukAB indeed does not follow the sequential binding paradigm of the other BCPFTs and exists as a dimer in solution [91].

Similar to leukocidins, the Hlg components bind sequentially to the cell membranes. While the receptors for gamma hemolysins have not been described, it has been shown that when human erythrocytes were first incubated with the F subunit and then washed extensively, addition of the S subunit induced rapid lysis of the cells [39,92]. These data suggested that in contrast to LukED and PVL, Hlg F subunit may be the primary receptor binding component on erythrocytes. On the other hand, it has been reported that LukS-PV and HlgC share the same receptor on PMNs, but the S components of other staphylococcal leukotoxins, HlgA, LukE, and LukM, do not compete with LukS-PV for its receptor [36]. More research is needed to clarify the nature of Hlg receptors, whether the two Hlgs (HlgAB and HlgCB) use the same receptors, and whether the paradigm of sequential binding of S followed by F will hold for gamma hemolysins.

## 5. Nine Subunits; How Many Toxins?

Currently, five S subunits (LukA, LukE, LukS-PV, HlgA, and HlgC) and four F subunits (LukB, LukD, LukF-PV, and HlgB) have been identified and their respective BCPFTs characterized as five functional toxins (LukAB, LukED, PVL, HlgAB, and HlgCB). We and others have reported that individual subunits from these different BCPFTs can form new functional toxins. Dalla Serra *et al.* reported that gamma hemolysin components can form mixed ABC toxins [82]. This cross combination is not limited to the family of hemolysins. We have recently shown that HlgB can form heterologous oligomers with LukS-PV [21]. Another report indicates that a combination of HlgA and LukF-PV is hemolytic towards rabbit red blood cells [81]. Siqueira *et al.* performed intravitreal injection of rabbits with six different combinations of S and F subunits from PVL and Hlg and compared these combinations based on ability to induce inflammation and necrosis [84]. This study showed various degrees of symptoms with the following order of severity: HlgA + LukF-PV > HlgAB ≥ LukS-PV + HlgB ≥ PVL > HlgCB, suggesting that a variety of new toxins with distinct potencies can be generated by these cross combinations.

**Figure 1:**
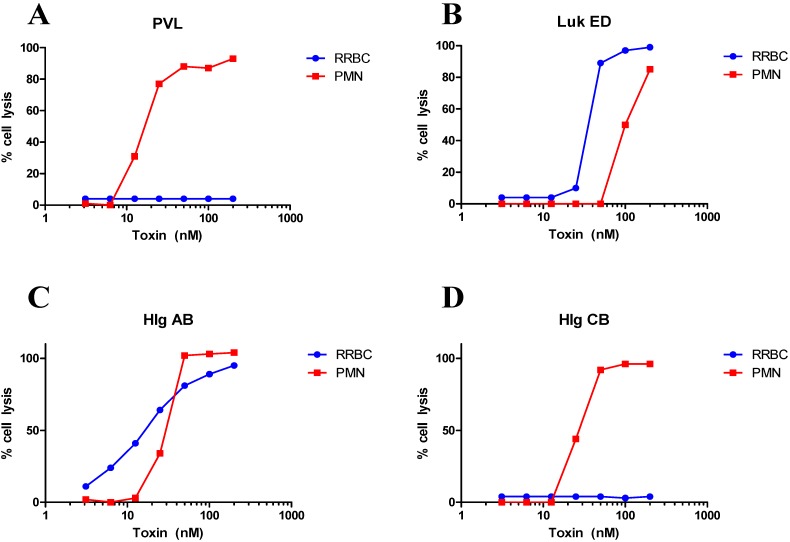
Comparison of hemolytic *versus* leukotoxic activities of (**A**) PVL; (**B**) LukED; (**C**) HlgAB, and (**D**) HlgCB. Dose response cytotoxicity assays were performed in HL-60 derived human PMNs or rabbit red blood cells (RRBC). Data are shown as percent cell lysis compared to no toxin control. Method: PMN Cytotoxicity assays were carried out as described previously [21] with minor modification. Briefly, DMSO (1.5%)-induced HL-60 cells (5 × 105 cells/well) were incubated with different homologous combinations of subunits for 24 h at 37 °C in an atmosphere of 5% CO_2_–95% air. The cell survival was measured after adding 100 ug/mL of XTT (Sigma–Aldrich, St. Louis, MO, USA) and further incubating cells for another 16 h. A colorimetric readout at OD470 nm is used to calculate % cell lysis. For the hemolysis assay, toxin combinations were incubated with 2% rabbit blood at 37 °C for 30 mins. The suspension was centrifuged and 100 uL of the supernatants were transferred into ELISA plate. Hemolysis was measured at OD416 nm.

The BCPFTs are best known for two distinct lytic activities, namely hemolysis towards red blood cells (hemolysins) and lysis of white blood cells (leukocidins). The distinction between hemolysins and leukocidins is largely based on initial approaches used during discovery of these toxins. However, hemolytic and leukocidal activities are not as distinct as previously believed. For example it is known that gamma hemolysins can lyse neutrophils [62,63,80]. As shown in Figure 1A, PVL appears to be the most potent lytic toxin towards PMN [80], but as previously reported this toxin has no detectable activity on rabbit erythrocytes. In contrast, LukED, known for its leukocidal activity, is also hemolytic to rabbit RBC [80] (Figure 1B). While HlgAB displayed equal activity towards erythrocytes and PMN (Figure 1C), HlgCB was as potent a leukocidin as PVL, as previously reported [62,63,80], but failed to lyse rabbit red blood cells (Figure 1D). These data indicate that the current nomenclature of BCPFTs as leukocidins *versus* hemolysins inaccurately reflects their breadth of functional activities.

**Figure 2:**
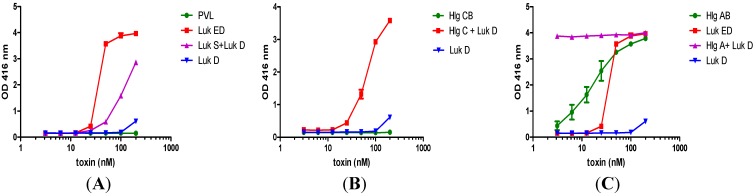
Cross combination of various leukotoxin subunits leads to qualitatively different toxin profiles in rabbit RBC hemolysis assay. OD_416_ reflects the degree of hemolysis (hemolysis method same as Figure 1). Non-canonical pairing of LukD conveys novel hemolytic activity to LukS-PV (**A**) and HlgC (**B**). Replacement of HlgB in HlgAB with LukD results in a toxin (HlgA+LukD) with substantially enhanced hemolytic activity (**C**).

Morinaga *et al.* have previously shown that S and F subunits between LukED and Hlg are largely interchangeable [80]. We further examined whether cross combinations can lead to qualitatively distinct toxic profiles. As shown in Figure 2, while PVL and HlgCB were not hemolytic, combination of either S subunit of these two toxins with LukD created a hemolytic toxin: LukS-PV/LukD (Figure 2A) and HlgC/LukD (Figure 2B). Replacement of HlgB by LukD in HlgAB also resulted in a new toxin (HlgA/LukD) with dramatically increased hemolytic activity that plateaued at a concentration of 3 nM (Figure 2C). Thus, it appears that qualitatively distinct toxins can be formed by cross combination of leukotoxin subunits. Furthermore, the fact that LukD, an F subunit, can confer novel hemolytic activity to an S subunit suggests that there may be cell type-specific direct interactions between the F subunit and components of erythrocyte plasma membrane. More research is needed to explore the various novel BCPFTs that can be generated through cross combination of the known subunits. Furthermore it is important to investigate if such cross combinations occur *in vivo*. Such studies can be significantly facilitated by development of subunit-specific monoclonal antibodies against the different leukotoxin components.

## 6. Cell Lysis *versus* Pro-inflammatory Activity

Phagocytes, primarily neutrophils, play a central role in the host defense against *S. aureus* and individuals with disorders of neutrophil function are highly susceptible to staphylococcal infections [93]. Thus it is not surprising that *S. aureus* has developed a large arsenal of immune modulatory molecules as well as pore-forming toxins to manage a complex set of interactions with the innate immune system, particularly the neutrophils. BCPFTs’ activities are not limited to cell lysis and involve a wide range of stimulatory and inhibitory activities with impact on the function of various cells of the innate immune system [47]. This dual mode of activity is not limited to BCPFTs and has been also described for PSM [94] and alpha hemolysin [95]. The BCPFTs can be utilized by the innate immune cells as danger signals to sense the invading bacteria. Binding of PVL to C5aR, the receptor for the powerful anaphylotoxin, C5a, may serve exactly this purpose by inducing proinflammatory responses in PMN. Indeed, Spaan *et al.* showed that PVL priming of neutrophils is dependent on binding to C5aR [38]. At the same time, binding of PVL to C5aR may block efficient activation of neutrophils by displacing the natural ligand. A similar mechanism has been described for the chemotaxis inhibitory protein of *S. aureus* (CHIPS) which also displaces the natural ligand of C5aR [96]. Consistent with this notion, LukS-PV inhibits the C5a-induced calcium mobilization in human neutrophils and a C5aR-transfected monocytic cell line [38].

PVL has been shown to be a strong inducer of IL-1β and inflammasome activation in primary human alveolar macrophages, a property that is synergistically enhanced by other cytolytic peptides such as delta hemolysin and PSM [76]. IL-1β in turn can trigger the release of chemotactic factors leading to massive neutrophil infiltration of the lung [76]. Graves *et al.* showed that sublytic concentrations of PVL induce PMN priming by enhancing the activity of N-formyl-methionyl-leucyl phenylalanine (fMLF) and redistribution of NADPH oxidase components, and this activity was proposed to enhance the host defense against *S. aureus* [97]. Studies reported by Malachowa *et al.*, showed strong inflammatory responses to PVL and LukAB (named LukGH in that study) in the skin of mice and rabbits and increased virulence of isogenic knockouts of LukAB and PVL in skin infection and bacteremia models. Contribution of PVL to host defense rather than virulence was also suggested by another report based on data with isogenic knockouts and polyclonal antibodies in a mouse model of skin abscess [26]. On the other hand, using isogenic knockouts in a rabbit model of USA300 necrotizing pneumonia, Diep *et al.* showed a key role for PVL in lung necrosis, pulmonary edema, alveolar hemorrhage and ultimately death [19]. Rabbits infected with USA300*Δpvl* showed significantly lower bacterial burden in lungs and higher survival rate compared to the wild-type counterpart [19]. PVL was also shown to increase the virulence of USA300 in a rabbit bacteremia model during the early stage of infection [20]. Lipinska *et al.* also showed that USA300 induced larger skin lesions and more necrosis than its isogenic PVL knockout in a rabbit model of dermonecrosis [23].

The discrepancy between various reports on the role of PVL in CA-MRSA pathogenesis may relate to different models used. As it is evident now through discovery of PVL receptor, PVL activity is restricted to humans and rabbits. Therefore, results obtained with isogenic knockouts in mice are difficult to decipher. Studies reported to date have not examined whether knocking out a specific BCPFT may impact the expression of other virulence factors, complicating the conclusions made solely based on knockouts. It is noteworthy that such a mechanism is not unprecedented, as it has been shown for toxic shock syndrome toxin-1 and staphylococcal enterotoxin B [98].

The response of human PMNs to LukED has been comprehensively analyzed using proteomic approaches [69]. This study shows secretion of 223 proteins and peptides by human PMN exposed to LukED. The secretions represented a diverse array of molecules including antimicrobial peptides, inflammatory proteins, enzymes, cytoskeleton proteins, and growth factors [69]. The secreted proteins were biologically active, thus suggesting that if the same response is triggered *in vivo*, it may prime neutrophils to orchestrate an inflammatory process as well as antimicrobial response. Malachowa *et al*. reported that similar to PVL, LukGH (LukAB) increases expression of CD11b on PMN surface but, unlike PVL, it fails to enhance fMLF-induced reactive oxygen production [74]. Treatment with PVL [99] or LukAB [74] is also reported to promote the release of neutrophil DNA and formation of extracellular traps, DNA webs containing antibacterial peptides that trap invading bacteria.

Collectively, these data indicate that the BCPFTs may represent a double-edged sword serving the host by being utilized as danger signals and the pathogen as a powerful tool of immune subversion. The balance between these two functions may be tilted in one or the other direction in different experimental models. Recognition of the toxins at sublytic concentrations as a danger signal can initiate a protective immune response. However, it is important to note that upon this priming and neutrophils recruitment to the site of infection, these important cells of innate defense are most likely exposed to lytic concentration of the toxin. BCPFTs may induce early priming but then inhibit functional consequences like phagocytosis which is critically dependent on the binding of several factors including C5a [100]. A massive inflammatory response can also lead to tissue damage and/or vascular injury as shown for PVL [19,101] and other *S. aureus* toxins such as alpha hemolysin [95,102,103]. Barrier disruption through tissue injury and vascular damage can in turn promote bacterial dissemination and metastatic growth in distant organs.

## 7. Leukotoxins as Targets for Prophylaxis and Immunotherapy

Several recent studies suggest a positive role for antibodies against PVL subunits and complications of *S. aureus* infection in humans. We recently performed a prospective study aimed at assessing whether pre-existing antibodies to staphylococcal toxins are associated with a lower risk of sepsis in adults with *S. aureus* bacteremia [104]. Serum samples were obtained from patients prior to or at presentation of *S. aureus* bacteremia to measure IgG antibody levels to 11 exotoxins. Of 100 eligible patients, 28 developed sepsis. Of the toxins tested, significant inverse correlation was observed between sepsis and antibody titers to alpha hemolysin, delta toxin, PSM, LukS-PV, and to a lesser extent, LukF-PV [104]. These findings are further supported by a report from Fritz *et al.* [105]. These authors correlated acute and convalescent serum antibody levels to Hla and PVL with the incidence of recurrent infection over 12 months in 235 children with *S. aureus* colonization, primary or recurrent SSTI, or invasive disease. Patients with invasive infections had the lowest pre-existing Hla and LukF titers but displayed the highest convalescent titers [105]. In contrast, another study by Hermos *et al.* showed that high levels of antibodies to PVL were not associated with resistance to *S. aureus* skin and soft tissue infections in a pediatric population [106].

Recently, we reported an attenuated form of LukS-PV with three mutations (T28F/K97A/S209A) that abrogate the ability of the toxin to oligomerize with LukF-PV or HlgB [21]. The mutant was fully attenuated when combined with wild-type LukF-PV in PMN cytotoxicity assays. The attenuated subunit vaccine was highly immunogenic and showed significant protection in a mouse model of *S. aureus* USA300 sepsis. Passive transfer experiments showed that the protection was mediated by antibodies to LukS-PV. Anti-LukS-PV polyclonal antibodies also inhibited the oligomerization of PVL toxin as well as cross oligomerization of LukS-PV with HlgB. Given that PVL does not bind to mouse C5aR [38], the protective efficacy observed in mice most likely reflects cross neutralization of other BCPFTS. Indeed, antisera to LukS-PV_T28F/K97A/S209A_ (denoted as LukS-Mut9) effectively neutralized the toxicity of purified HlgCB and supernatants of the PVL negative strain 8325-4 towards human PMN [21].

**Figure 3:**
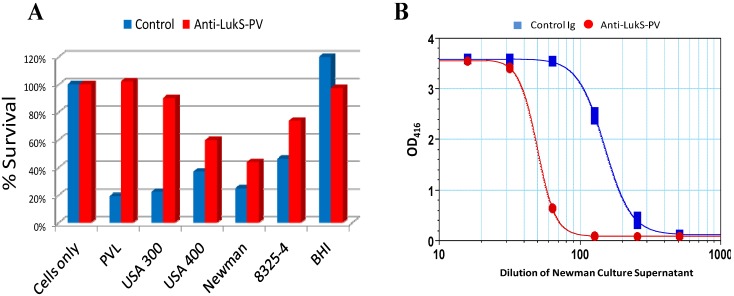
(**A**) Neutralization of PMN cytotoxic activity in supernatants of PVL positive (USA300 and USA400) and PVL negative (Newman and 8325-4) strains by antibodies to LukS-PV; (**B**) Neutralization of hemolytic activity of *S. aureus* strain Newman by antibodies to LukS-PV. Method: PMN cytotoxicity assays were carried out as described in Figure 1. For PMN TNA, purified rabbit anti-LukS IgG were incubated with serially diluted bacterial culture supernatant (grown O/N in BHI and filter sterilized) as a source of toxins. The mixtures were incubated for 1 hour at 37 °C in an atmosphere of 5% CO_2_–95% air. Then, DMSO (1.5%)-induced HL-60 cells (5 × 105 cells/well) were added and incubated for 24 h at 37 °C. Cell survival was measured as described previously (Figure 1). For hemolysis TNA assay, purified rabbit anti-LukS IgG/control IgG were incubated with serially diluted Newman culture supernatant (grown O/N in BHI and filter sterilized) as a source of toxins for 10 mins at room temperature. Two percent rabbit blood was added and further incubated at 37 °C for 30 mins. The suspension was centrifuged and 100 uL of the supernatants were transferred into the ELISA reading plate. Hemolysis was measured as described in Figure 1.

Given the diversity of *S. aureus* BCPFTs, an effective vaccine must be able to induce broadly neutralizing antibodies to major members of this family. PVL, Hlg and LukED show a high degree of sequence identity (>70%) within each subunit class, whereas lower (~30%) sequence identity is observed between the S and F subunits (Table 2). The most divergent leukotoxin is LukAB with sequence identity of 33%–40% within each class with other members of BCPFT family. Based on this sequence identity and the breadth of functional activities of these toxins, it is possible that vaccine and immunotherapeutic intervention strategies can be devised that provide broad neutralizing activity. Consistent with this hypothesis we found that antibodies raised against LukS-PV neutralize leukotoxic activity towards human PMN of PVL-negative *S. aureus* strains (Figure 3A). Anti-LukS-PV also reduced the hemolytic activity in the culture supernatants of the PVL-negative strain Newman (Figure 3B). These data suggest that broad neutralization of the BCPFTs is feasible. An exception may be LukAB due to its low sequence identity with other family members.

Leukotoxins also represent a potential target for immunotherapy. Neutralizing antibodies to leukotoxins combined with monoclonal antibodies to Hla and possibly other toxins such as superantigens could be a viable therapeutic product for treatment of life threatening conditions such as ventilator-associated pneumonia. Recently, Laventie *et al.* reported heavy chain only monoclonal antibodies (HCAb) targeting LukS-PV and LukF-PV [107]. The investigators also generated a tetravalent bispecific antibody that targets both subunits. The antibodies protected neutrophils from PVL-induced lysis as well as induction of inflammatory response. Importantly, the anti-PVL antibodies showed cross neutralization of HlgCB. The efficacy of the HCAbs was demonstrated in a noninfectious endophtalmitis model in rabbits.

**Table 2:** Sequence identity (%) between members of *S. aureus* BCPFTs.

Class	Subunit	S-Subunit					F-Subunit			
		LukS-PV	Hlg C	Hlg A	Luk E	Luk A	LukF-PV	Hlg B	Luk D	Luk B
S-Subunit	LukS-PV	100	82	68	71	33	30	28	31	27
	Hlg C		100	70	70	34	30	28	30	27
	Hlg A			100	70	35	29	29	31	28
	Luk E				100	36	28	29	30	27
	Luk A					100	28	28	30	31
F-Subunit	LukF-PV						100	72	82	39
	Hlg B							100	76	40
	Luk D								100	39
	Luk B									100

For an effective immunotherapy, it is important that candidate immunoprotectants show broad neutralization activity. Given the high degree of sequence identity within the leukotoxins (except for LukAB) and the fact that polyclonal [21] and monoclonal [107] cross neutralization between PVL and HlgCB has been demonstrated, it is conceivable that such broadly neutralizing monoclonal antibodies would be feasible at least for PVL, LukED, and Hlgs. A monoclonal antibody cross neutralizing these toxins along with LukAB is unlikely. However, bispecific antibodies could be generated covering the entire leukotoxin family. Such monoclonals could be an important component of a future multivalent antibody combination for treatment of life-threatening *S. aureus* complications. Protective efficacy has been shown in mouse models against dermocecrosis, pneumonia, or sepsis with antibodies to Hla [108,109,110] or superantigens [111]. Such antibodies could be additional components of a combination therapy.

## 8. Concluding Remarks

Recent discovery of the receptors for various leukotoxins has significantly expanded our understanding of the functional and species specificities of the bicomponent pore-forming toxins. Collectively, these data indicate that these toxins should be given serious consideration as targets for preventive vaccination or immunotherapeutic intervention. The history of failures with single subunit vaccines for *S. aureus* teaches us that a successful vaccine for this pathogen must be multivalent, targeting multiple virulence strategies; inducing functional antibodies; and most likely also inducing effective Th17 T cell responses and interleukin 17. Toxoids based on BCPFTs could be an important component of such a multivalent approach. Given the diversity of the leukotoxins, the BCPFT vaccine must be able to induce broadly neutralizing antibodies. In light of the potential of these toxins to act as a double-edged sword, caution should be taken in analysis of safety of these vaccines in the context of an ongoing *S. aureus* infection in relevant animal models, such as rabbits, before transitioning into the clinic. It remains to be determined whether a multivalent vaccine solely based on toxins will be sufficient to prevent *S. aureus* infections or ameliorate complications, or if such a toxoid vaccine needs to be complemented with key cell surface targets involved in establishment of infection.
